# Unique [^18^F]FDG PET imaging pattern of drug-induced acute generalized exanthematous pustulosis within the SCAR-spectrum

**DOI:** 10.1007/s00259-022-06060-9

**Published:** 2022-12-09

**Authors:** E. Novruzov, D. Schmitt, Y. Mori, J. Kirchner, G. Kobbe, J. Reifenberger, E. Mamlins, C. Antke, F. L. Giesel

**Affiliations:** 1 Department of Nuclear Medicine, Medical Faculty, Heinrich-Heine-University, University Hospital Dusseldorf, Dusseldorf, Germany; 2 Department of Diagnostic and Interventional Radiology, Medical Faculty, Heinrich-Heine-University, University Hospital Dusseldorf, Dusseldorf, Germany; 3 Department of Hematology, Oncology and Clinical Immunology, Medical Faculty, Heinrich-Heine-University, University Hospital Dusseldorf, Dusseldorf, Germany; 4 Department of Dermatology, Medical Faculty and University Hospital Düsseldorf, Heinrich-Heine-University Düsseldorf, Düsseldorf, Germany

Severe cutaneous adverse reactions (SCARs) including acute generalized exanthematous pustulosis (AGEP) are usually medication-related and represent a rare, heterogeneous group of T-cell-triggered delayed hypersensitivity reactions resulting from interactions between small molecule drugs, human leucocyte antigen (HLA) Class I molecules, and T-cell receptors (TCR) [1–5].

A 62-year-old male patient with an anaplastic large T-cell lymphoma in medical history presented in our clinic with an abrupt onset of generalized maculopapular exanthema without mucosal involvement after initiating antibiotic therapy with a combination of caspofungin, vancomycin, and meropenem due to community-acquired pneumonia. A whole-body [^18^F]FDG PET/CT scan, performed four days after initial symptoms, demonstrated a very striking pattern of uniformly increased metabolic uptake throughout the skin. A marked [^18^F]FDG uptake was observed in the spine and spleen (SUV_max_: 7,4) due to systemic inflammatory response. Moreover, the neck and groin areas exhibited also an intense tracer uptake (SUV_max_: 7,3) mainly due to a partial volume effect and, to a lesser extent, physical stress in the skinfold areas in addition to the abovementioned dermatological changes. The elevated [^18^F]FDG uptake at the injection site in the right wrist is also to be mentioned. Skin biopsy revealed a superficial perivascular dermatitis with infiltration of the outermost layer of the dermis by lymphocytes, histiocytes, and single plasma cells, accompanied by focal parakeratosis and minimal spongiotic loosening of the stratum corneum (e).

To our best knowledge, this is the first metabolic imaging of SCAR syndrome, in particular meropenem-induced AGEP, using [^18^F]FDG PET/CT scan.


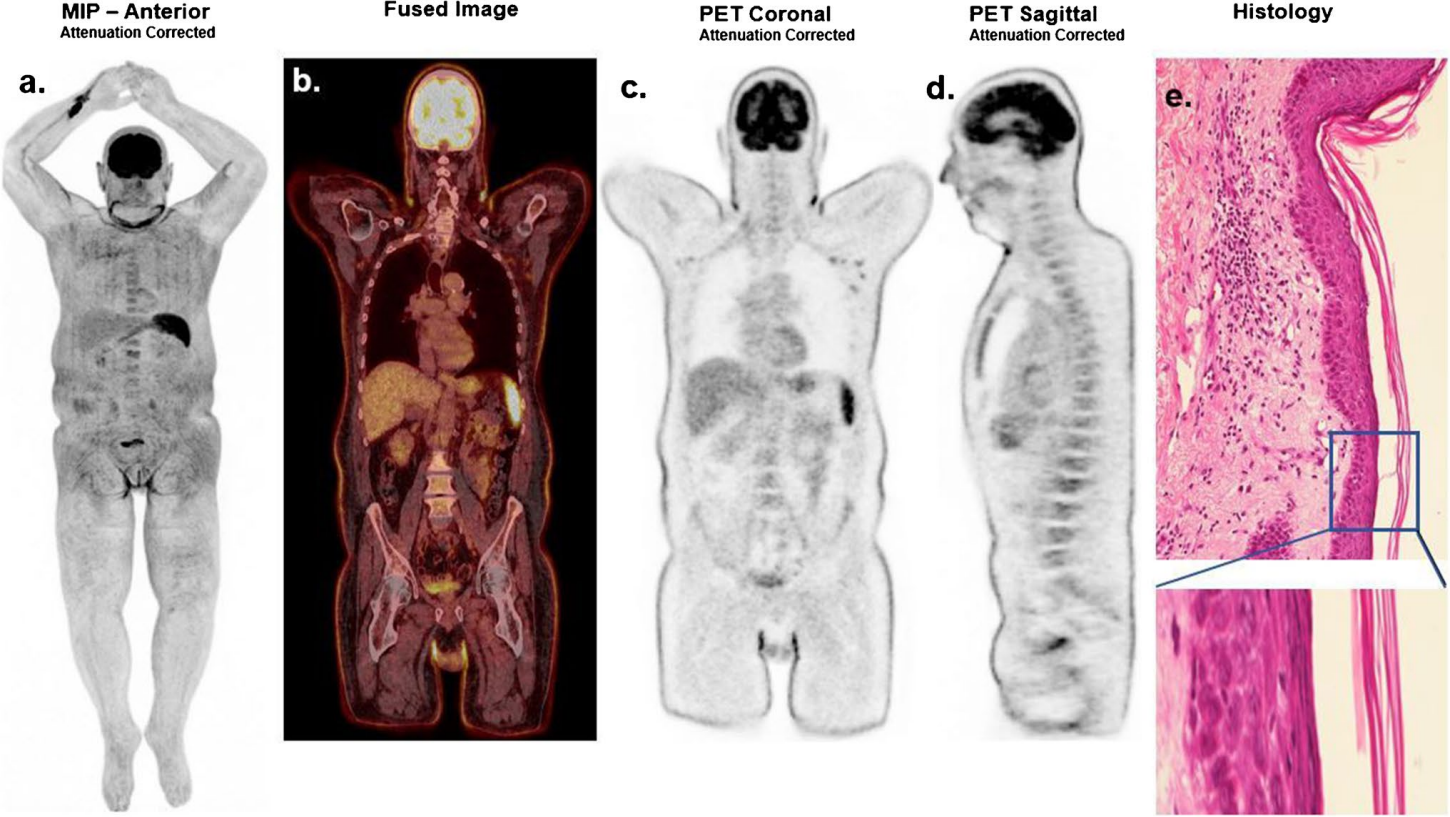


## Declarations

All procedures were in accordance with the ethical standards of the institutional and/or national research committee and with the 1964 Helsinki Declaration and its later amendments or comparable ethical standards. For case reports from clinical practice, no trial registration is required.
